# Human enteric adenovirus F40/41 as a major cause of acute gastroenteritis in children in Brazil, 2018 to 2020

**DOI:** 10.1038/s41598-022-15413-1

**Published:** 2022-07-02

**Authors:** Lilian Gonçalves do Nascimento, Alexandre Madi Fialho, Juliana da Silva Ribeiro de Andrade, Rosane Maria Santos de Assis, Tulio Machado Fumian

**Affiliations:** Laboratory of Comparative and Environmental Virology, Oswaldo Cruz Institute, Oswaldo Cruz Foundation, Rio de Janeiro, RJ CEP 21045-900 Brazil

**Keywords:** Gastroenteritis, Infection, Molecular medicine, DNA sequencing

## Abstract

Human adenovirus (HAdV) types F40/41 have long been recognized as major viral agents of acute gastroenteritis (AGE) in children. Despite this, studies on HAdV molecular epidemiology are sparse, and their real impact is likely under-estimated. Thus, our goal was to investigate HAdV incidence, enteric and non-enteric types circulation, co-detections with rotavirus and norovirus and DNA shedding in stool samples from inpatients and outpatients from eleven Brazilian states. During the three-year study, 1012 AGE stool samples were analysed by TaqMan-based qPCR, to detect and quantify HAdV. Positive samples were genotyped by partial sequencing of the hexon gene followed by phylogenetic analysis. Co-detections were accessed by screening for rotavirus and norovirus. Overall, we detected HAdV in 24.5% of single-detected samples (n = 248), with a prevalence of type F41 (35.8%). We observed a higher incidence in children between 6 to 24 months, without marked seasonality. Additionally, we observed a statistically higher median viral load among single-detections between enteric and non-enteric types and a significantly lower HAdV viral load compared to rotavirus and norovirus in co-detections (p < 0.0001). Our study contributes to the knowledge of HAdV epidemiology and reinforces the need for the inclusion of enteric types F40/41 in molecular surveillance programs.

## Introduction

Acute gastroenteritis (AGE) remains a leading cause of morbidity and mortality in low- and middle-income countries, and despite the continuous improvement in children’s public health worldwide, AGE still figures as a major cause of death in children under 5 years old^1,2^. Among the gastroenteric viruses, rotavirus A, norovirus and human adenovirus (HAdV) are the leading causes of diarrhea worldwide^3–5^. Furthermore, through mathematical modeling, the GBD Study estimated that enteric HAdV (types F40/41) were responsible for 52,613 deaths (95% uncertainty interval) among children younger than 5 years old, behind only rotavirus and *Shigella*^2^.

HAdV belongs to the *Adenoviridae* family (genus *Mastadenovirus*) and are large (∼90 nm), non-enveloped viruses with a double-stranded linear DNA genome ranging from 26 to 45 kb in size. Its icosahedral capsid is composed of three major proteins: hexon, penton base and fiber, along with minor capsid proteins arranged in a complex architecture that surrounds the inner DNA-containing core and its associated proteins^6^. To date, 111 types of HAdV have been characterized and further divided into seven species (A–G) (http://hadvwg.gmu.edu/). Initially, serological approaches were applied for HAdV classification and, currently, new types are characterized using molecular methods and bioinformatics tools^7^.

Depending on the tissue tropism, HAdV types can cause a wide range of clinical symptoms, usually mild and self-limited, often associated with respiratory, gastrointestinal (GI) or conjunctival diseases. Less frequently, HAdV can affect other systems, such as the urinary tract, liver, pancreas, cardiovascular system and even the central nervous system^8^. HAdV can cause severe illness and often requires hospitalization, with immunocompromised individuals and the pediatric population at higher risk of disease severity^9–13^.

It has been almost 40 years since enteric types F40/41 were identified as significant AGE agents^14–16^. Previous studies with HAdV F40/41 have shown that modifications in major and minor proteins responsible for the organization of the capsid structure, as well as the presence of two fiber proteins, differently from non-enteric types, could explain their stability and tropism on the GI tract^17–20^.Two comprehensive studies of diarrheal etiology performed between 2007 and 2014 in several low- and middle-income countries demonstrated the high attributable incidence (AI) and the burden of disease caused by enteric HAdV^12,21^. In both studies, HAdV types F40/41 had the second-highest AI among infants, after rotavirus.

Non-enteric types are frequently identified in stool samples from patients with AGE, with some studies reporting detections of non-enteric HAdV, such as HAdV-12 (species A) and HAdV-52 (species G), as the only enteric agents linked with AGE outbreaks^13,22–25^. However, whether these types can be linked as AGE causative agents remains unclear.

In Brazil, the rotavirus vaccine (RV1, Rotarix) was implemented into the National Immunization Program in 2006, and rotavirus-associated infections, hospitalizations and deaths has been declining substantially^26^. Two previous studies from our group have recently demonstrated the epidemiological features of rotavirus and the high incidence and molecular epidemiology of norovirus from AGE cases in Brazil^27,28^. There are few studies in Brazil and elsewhere in regards to HAdV incidence, epidemiology and molecular characterization, especially in the post-rotavirus vaccine era. Therefore, our aim was to investigate the incidence of HAdV infections in AGE cases over a 3-year period (2018–2020) in eleven Brazilian states. We also tested HAdV in rotavirus- and norovirus-positive samples to evaluate the frequency of co-detection with these major AGE pathogens. By qPCR, we determined HAdV viral loads to understand differences in DNA shedding among enteric and non-enteric types, co-detections and age groups. Additionally, HAdV types were genetically characterized by sequencing a conserved region of the hexon gene to assess their molecular epidemiology.

## Results

### HAdV epidemiology

**Figure 1:**
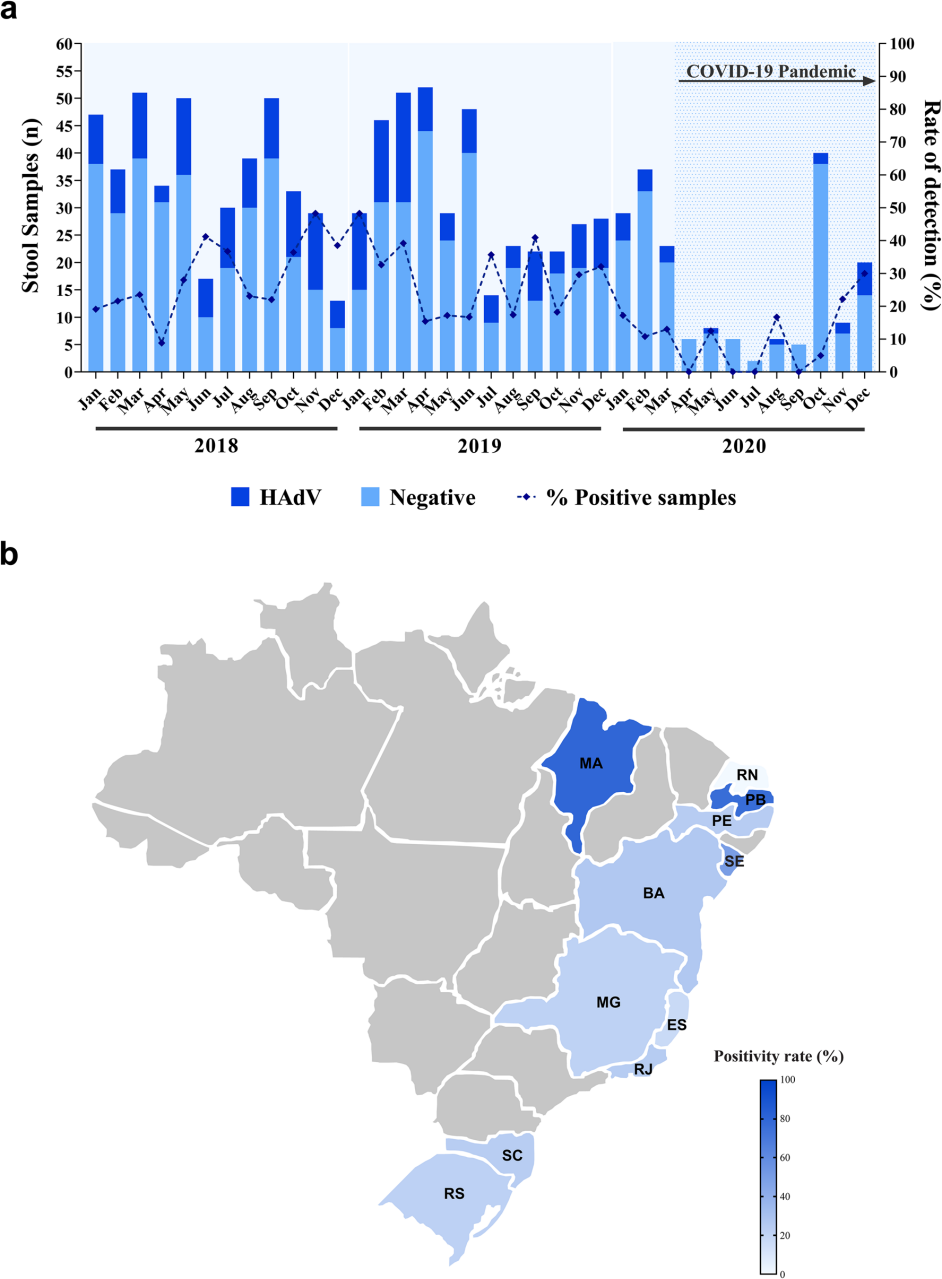
**(a)** Monthly distribution of tested acute gastroenteritis samples, HAdV-positive samples and detection rates in Brazil, 2018–2020. **(b)** Map of Brazil highlighting HAdV detection rates in each of the eleven states included in this three-year study. This figure was designed in Adobe Illustrator 26.0 (https://www.adobe.com/).

During the three-year study period (2018–2020), we tested a total of 1,012 stool samples from symptomatic inpatients and outpatients with AGE that had previously tested negative for rotavirus and norovirus. HAdV was detected in 24.5% of samples (n = 248), being 26.7% (115/430) in 2018, 27.9% (109/391) in 2019 and 12.6% (24/191) in 2020 (Table 1). HAdV monthly detection rates varied from 5% (October 2020) to 48.3% (November 2018 and January 2019) (Fig. 1a). A slightly higher detection rate was observed in 2019 compared to 2018, but without statistical significance (*p* = 0.7158). In contrast, the detection rate of HAdV in 2020 was significantly lower compared to 2018 and 2019 (*p* < 0.0001). Figure 1b shows HAdV-positivity rate for each of the 11 Brazilian states included in our study.

**Table 1:** Number of HAdV-positive fecal samples identify through laboratory-based surveillance by region and state in Brazil from 2018 to 2020, among rotavirus- and norovirus- negative samples.

Region/State	No. of fecal samples—positive/tested (%)				*p*-value^1^ (Chi-square test)			
	2018	2019	2020	Total	2018 vs. 2019	2018 vs. 2020	2019 vs. 2020	Total
Northeastern	44/123 (35.8)	29/73 (39.7)	10/64 (15.6)	83/260 (31.9)	0.5799	0.0039	0.0018	0.0047
Bahia	21/70	17/47	10/63					
Paraíba	13/17	0/0	0/0					
Pernambuco	3/17	5/13	0/0					
Rio Grande do Norte	0/0	0/4	0/1					
Sergipe	3/11	7/9	0/0					
Maranhão	4/5	0/0	0/0					
Southeastern	26/122 (21.3)	35/141 (24.8)	5/49 (10.2)	66/312 (21.2)	0.5011	0.0883	0.0306	0.0972
Espírito Santo	6/33	11/65	0/4					
Minas Gerais	6/37	5/17	4/17					
Rio de Janeiro	14/52	19/59	1/28					
Southern	45/185 (24.3)	45/177 (25.4)	9/78 (11.5)	99/440 (22.5)	0.8088	0.0190	0.0124	0.0370
Rio Grande do Sul	26/102	19/91	8/53					
Santa Catarina	19/83	26/86	1/25					
Total	115/430 (26.7)	109/391 (27.9)	24/191 (12.6)	248/1012 (24.5)	0.7158	< 0.0001	< 0.0001	0.0001

^1^*p*-values were calculated by comparing the frequency of HAdV detection between the three years of the study (Total) and between each year for each region.

Regarding regional analysis, higher HAdV detection rate was observed in the Northeastern region (31.9%) compared to the Southeastern and Southern regions (21.2% and 22.5%, respectively). HAdV detection rates were significantly higher in 2018 compared to 2020 in the Northeastern (*p* = 0.0039) and Southern regions (*p* = 0.0190), as well as in 2019 compared to 2020 in all regions (Northeastern, *p* = 0.0018; Southeastern, *p* = 0.0306; and Southern, *p* = 0.0124). In contrast, over the first two years of the study (2018 and 2019), HAdV detection rates in all three regions were similar and not statistically significant. Additionally, over the three-year study period, the two Southern states (Santa Catarina and Rio Grande do Sul) accounted for nearly 40% of all HAdV-positive samples, whereas the state of Bahia accounted for more than half of HAdV-positive samples from the Northeastern region (Table 1).

Except for four months in 2020, we detected HAdV year-round without any marked seasonality. We observed a higher positivity of HAdV during the spring and summer months than in autumn and winter months, although not statistically significant (*p* = 0.1651) (Table 2). We also investigate HAdV seasonal circulation pattern among the regions since Brazilian Northeastern and Southeastern regions are tropical areas and Southern region has a subtropical climate pattern. In the Northeastern region, HAdV was detected at significantly higher rates during spring and summer months than in autumn and winter months (*p* = 0.0306), with higher detection rates in November and December months. Table 2 shows the distribution of enteric and non-enteric HAdV types for each Brazilian region.

**Table 2:** Distribution of enteric and non-enteric HAdV types and seasonal distribution of HAdV-positive samples for each Brazilian region from 2018 to 2020.

Region	HAdV-positive		No. of fecal samples—positive/tested (%)				*p*-value (Chi-square test)
	Enteric	Non-enteric	Summer and Spring		Autumn and Winter	Total	
Northeastern	17	16	62/108 (36.5)		21/69 (23.3)	83/173 (31.9)	0.0306
Southeastern	14	9	27/124 (17.9)		39/122 (24.22)	66/312 (21.2)	0.1704
Southern	14	30	55/173 (24.1)		44/168 (20.8)	99/440 (22.5)	0.03979
Total	45	55	144/405 (26.2)		104/463 (22.5)	248/1012 (24.5)	0.1651

HAdV was detected across all age groups, with detection rates varying from 37.4% (77/206) in children aged between 12 and 24 months old to 8.9% (21/235) in adults over 20 years old. HAdV detection rate was significantly higher among children in the age group of > 12–24 months old compared to the other age groups, except for the age groups of > 6–12 months and > 24–36 months old (Table 3). More than half of the stool samples analyzed (68.6%) belonged to children under five years old. Regarding adults included in the group over 20 years old, we detected HAdV in 9.6% (19/198) and 5.4% (2/37) of samples from the age groups of > 20–59 years and > 60 years old, respectively. As for gender, HAdV detection rates were similar between females (24.4%, 112/458) and males (24.7%, 136/550) (*p* = 0.9201).

**Table 3:** Number of tested and HAdV-positive fecal samples through laboratory-based surveillance by age group in Brazil during 2018–2020.

Age group	No. of fecal samples—positive/tested (%)				*p*-value^1^ (Chi-square test)
	2018	2019	2020	Total	
0–6 m	19/88 (21.6)	14/60 (23.3)	3/26 (11.5)	36/174 (20.7)	0.004
> 6–12 m	21/61 (34.4)	24/55 (43.6)	4/23 (17.4)	49/139 (35.3)	0.6874
> 12–24 m	38/92 (41.3)	34/85 (40.0)	5/29 (17.2)	77/206 (37.4)	–
> 24–36 m	22/58 (37.9)	19/54 (35.2)	4/21 (19)	45/133 (33.8)	0.5068
> 36–60 m	2/14 (14.3)	3/16 (18.8)	3/12 (25)	8/42 (19)	0.0225
> 60 m–19 y	5/29 (17.2)	6/34 (17.6)	1/20 (5)	12/83 (14.5)	0.0001
> 20 y	8/88 (9.1)	9/87 (10.3)	4/60 (6.7)	21/235 (8.9)	< 0.0001

*m* months old, *y* years old.

^1^*p*-values were calculated between the age group of > 12–24 m and each other.

### Co-detection, genetic characterization and viral load

To investigate HAdV co-detections, we randomly selected ~ 35% of rotavirus- and norovirus-positive samples over the period. We tested HAdV in a total of 223 samples and 30% (67/223) of these were positive. Although not statistically significant (*p* = 0.519), HAdV was more frequently detected in rotavirus-positive samples (33.3%, 21/63) than in norovirus-positive samples (28.8%, 46/160). Additionally, HAdV was more prevalent in the pool of rotavirus- and norovirus-positive samples (30%) than among negative samples for both viruses (24.5%), but without statistical significance (*p* = 0.090).

Regarding age distribution among the co-detections, the highest detection rate of HAdV was observed in the age strata of > 12–24 months old, for both rotavirus (50%) and norovirus (39.1%). Moreover, we detected HAdV in at least 29% of rotavirus- and norovirus-positive samples in children under the age of five. HAdV was significantly less frequently detected among samples from patients older than five years old (*p* = 0.0039), including children, adolescents and adults. Additionally, no HAdV co-detection was observed in samples from adults older than 60 years old (n = 7) (Table 4).

**Table 4:** Number of rotavirus and norovirus-positive samples tested to assess HAdV-positive frequency among co-detected samples through laboratory-based surveillance by age group in Brazil during 2018–2020.

Age group (months)	No. of fecal samples—positive/tested (%)			*p*-value^1^ (Chi-square test)
	Rotavirus + HadV	Norovirus + HAdV	Total	
0–6	3/7 (42.9)	6/18 (33.3)	9/25 (36)	0.5972
> 6–12	2/6 (33.3)	10/35 (28.6)	12/41(29.3)	0.1948
> 12–24	3/6 (50)	18/46 (39.1)	22/52 (40.4)	–
> 24–60	8/22 (36.4)	7/25 (28)	15/47 (31.9)	0.2858
> 60	5/22 (22.7)	5/36 (13.9)	10/58 (17.2)	0.0039

^1^*p*-values were calculated between the age group of > 12–24 months and each other.

**Figure 2:**
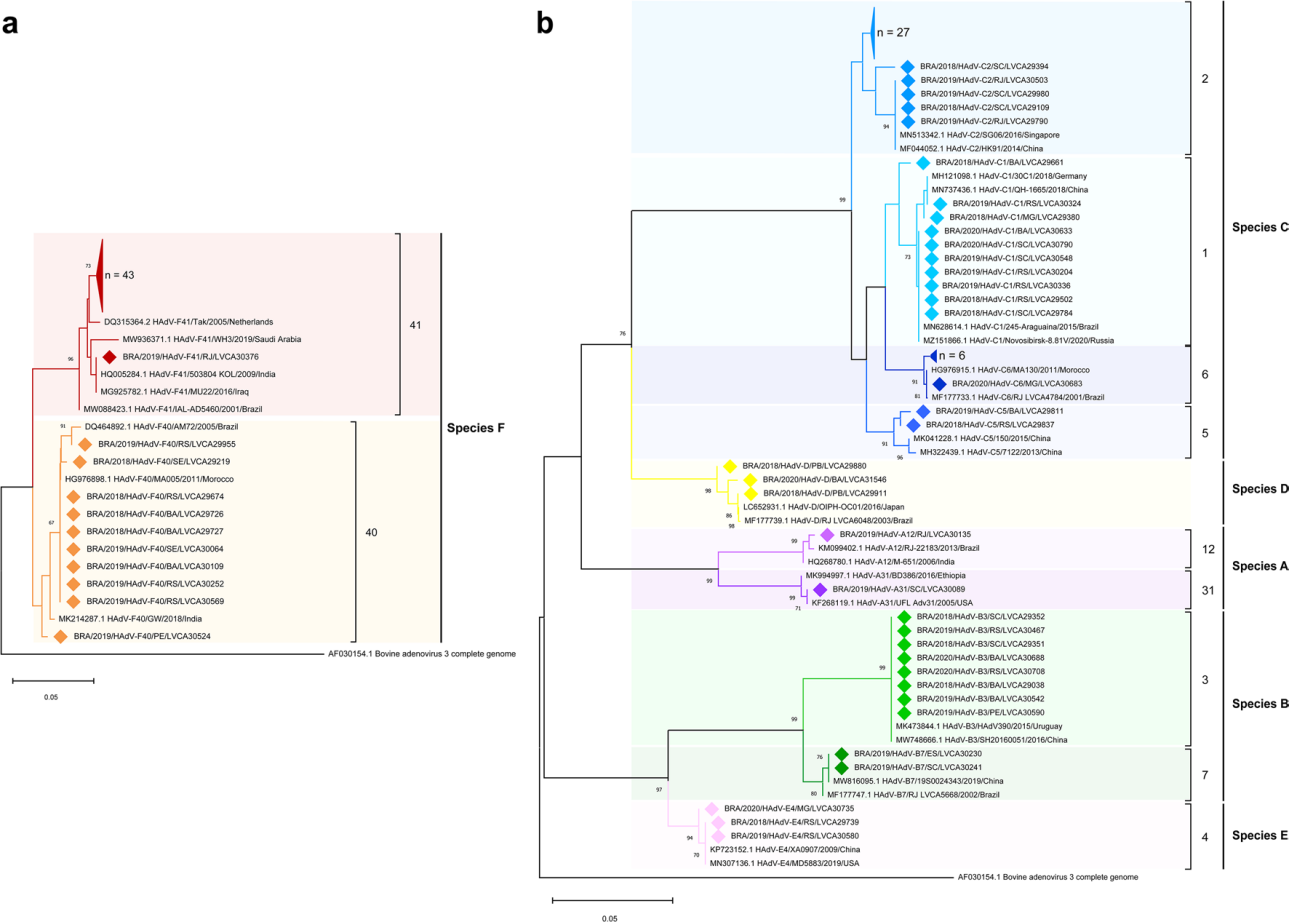
Phylogenetic analyses based on a conserved region of the hexon gene nucleotide (nt) sequence (located at positions 21–322, upstream of the surface loop l_1_) of the circulating Brazilian **(a)** enteric and **(b)** non-enteric Human Adenovirus (HAdV) types. Reference strains were downloaded from GenBank and labeled with their accession number followed by type, register number, year and country. Strains obtained (marked with a diamond) are shown as per country followed by type, year of collection, state and the LVCA internal register number (i.e., BRA/2019/HAdV-C2/RJ/LVCA30503). Neighbour-joining phylogenetic tree was constructed with MEGA X software and bootstrap tests (2000 replicates) based on the Kimura two-parameter model. Bootstrap values above 60% are given at branch nodes.

We successfully sequenced 39% of samples (123/315), being 44 from 2018, 65 from 2019 and 14 from 2020. Phylogenetic analyses of the partial portion of the hexon gene showed the circulation of 12 HAdV types, belonging to six species (A, B, C, D, E and F) (Fig. 2). HAdV enteric types were the most predominant genotypes (43.9%; n = 54), with higher detection of type F41 (n = 44) compared to type F40 (n = 10). HAdV species C, was the second most predominant (39%), characterized as HAdV-2 (26%), -1 (5.7%), -6 (5.7%), and -5 (1.6%). Species B was detected in 8.1% of samples, with detection of HAdV-3 in 6.5% and -7 in 1.6% of samples. HAdV species E (type 4) was detected in 2.4% of samples, and species A was detected in two samples (1.6%), belonging to types 12 and 31 (Fig. 2). Species D was detected in 2.4%, however, due to the high similarity of the amplified portion of the hexon gene within this species, D-types could not be defined.

**Figure 3:**
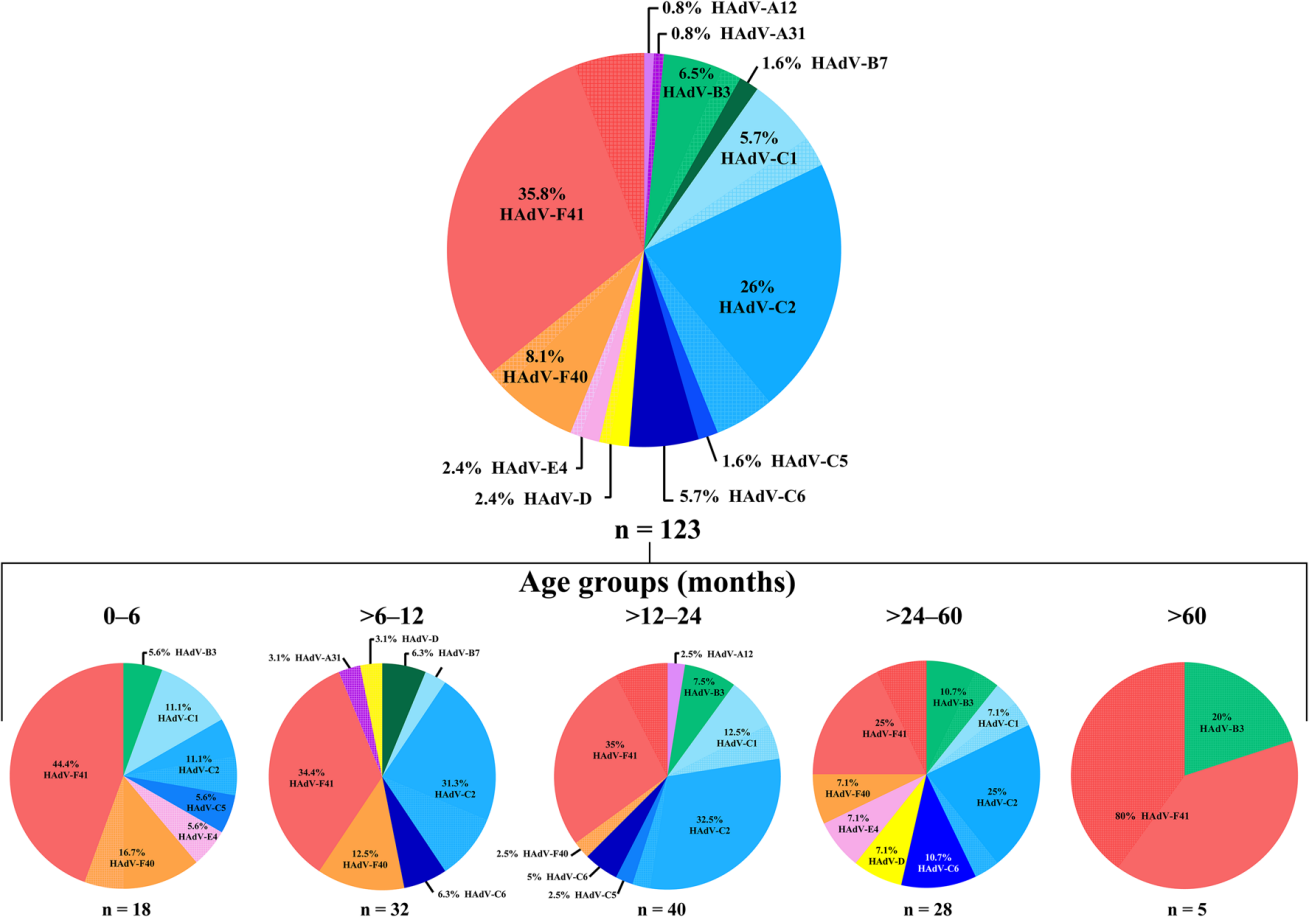
The total frequency of HAdV types obtained from sequenced samples and types percentage distribution by age groups. HAdV types obtained from co-detections with rotavirus or norovirus are represented by a crosshatched pattern.

Regarding type distribution across age groups, HAdV F40/41 were the most frequently detected in all age groups, except for the age group > 12–60 months old, where species C predominated, specifically HAdV-C2. Detailed analyses of types distribution by age groups are shown in Fig. 3.

**Figure 4:**
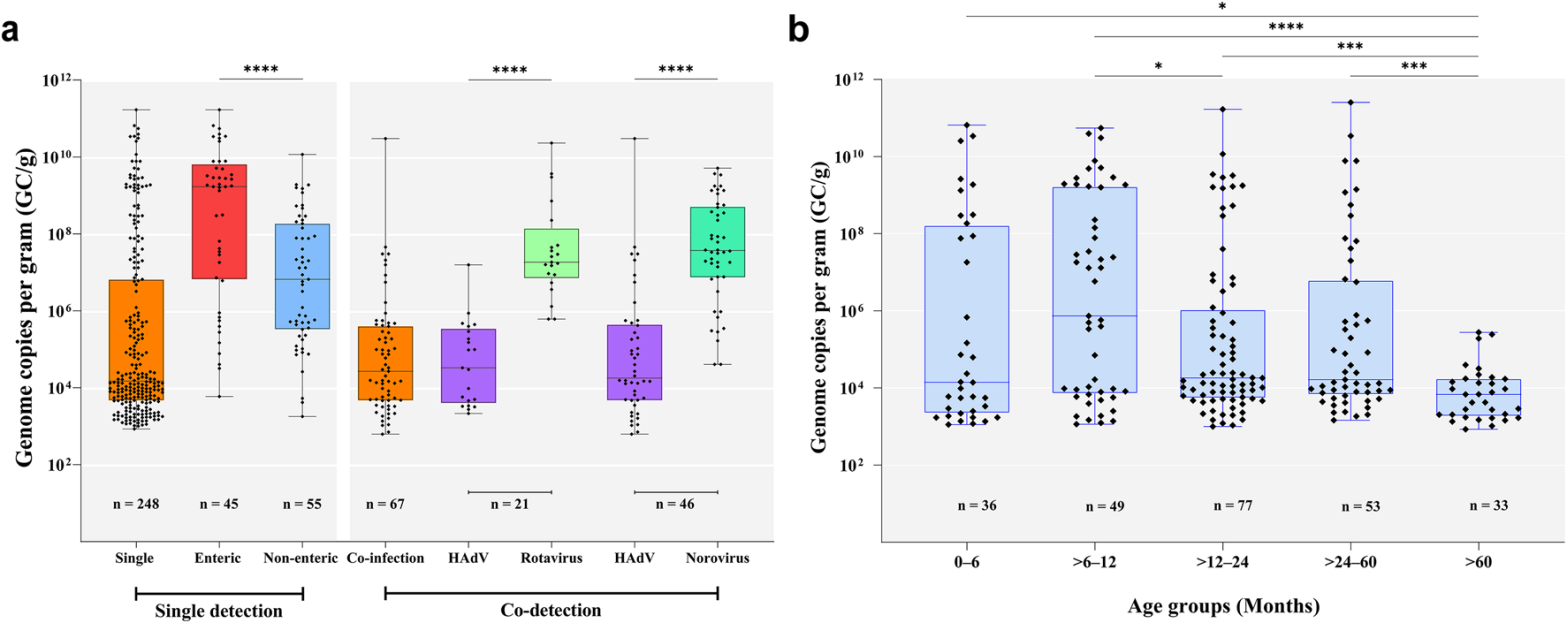
**(a)** Enteric viruses viral load expressed as genome copies per gram of stool (GC/g). Among HAdV single- and co-detected samples with rotavirus and norovirus. **(b)** HAdV single-detections viral load among different age groups in Brazil, 2018–2020. Box-and-whisker plots show the first and third quartiles (equivalent to the 5th and 95th percentiles), the median (the horizontal line in the box), and range of HAdV viral load concentrations. *p ≤ 0.05; ***p ≤ 0.001; ****p ≤ 0.0001.

We also investigated HAdV fecal shedding among different HAdV types, co-detections and age groups. We found significantly higher HAdV DNA loads in samples characterized as enteric types (F40/41) compared to non-enteric types (*p* < 0.0001). DNA viral loads of enteric and non-enteric types ranged from 8.5 × 10^2^ to 1.7 × 10^11^ GC/g (median of 1.7 × 10^9^) and 1.8 × 10^3^ to 1.2 × 10^10^ GC/g (median of 6.6 × 10^6^ GC/g), respectively (Fig. 4a). In regards to age groups, we found significantly lower HAdV fecal viral loads in the age group of > 60 months old compared to the other age stratas. The age group of > 6–12 months old had the highest median viral load (7.4 × 10^5^ GC/g) among all ages, whereas the age strata of > 60 months old had the lowest median viral load (6.9 × 10^3^ GC/g) (Fig. 4b).

Comparing viral shedding in HAdV-positive co-detected samples, we found significantly higher viral loads of rotavirus or norovirus compared to HAdV viral loads (*p* < 0.0001). The median viral load for HAdV and rotavirus in co-infected samples was 3.1 × 10^4^ and 2.2 × 10^7^ GC/g, respectively, meanwhile for HAdV and norovirus co-detections was 1.5 × 10^4^ and 3.7 × 10^7^ GC/g, respectively (Fig. 4a). HAdV viral loads ranged from 8.5 × 10^2^ to 1.7 × 10^11^ GC/g (median value of 1.5 × 10^4^) in single detected samples, and from 6.2 × 10^2^ to 3 × 10^10^ GC/g (median value of 1.6 × 10^5^) in co-detected samples (Fig. 4a). Although not statistically significant (*p* = 0.545), we observed a wider variation range of DNA concentration in single-detected than in co-detected samples.

**Figure 5:**
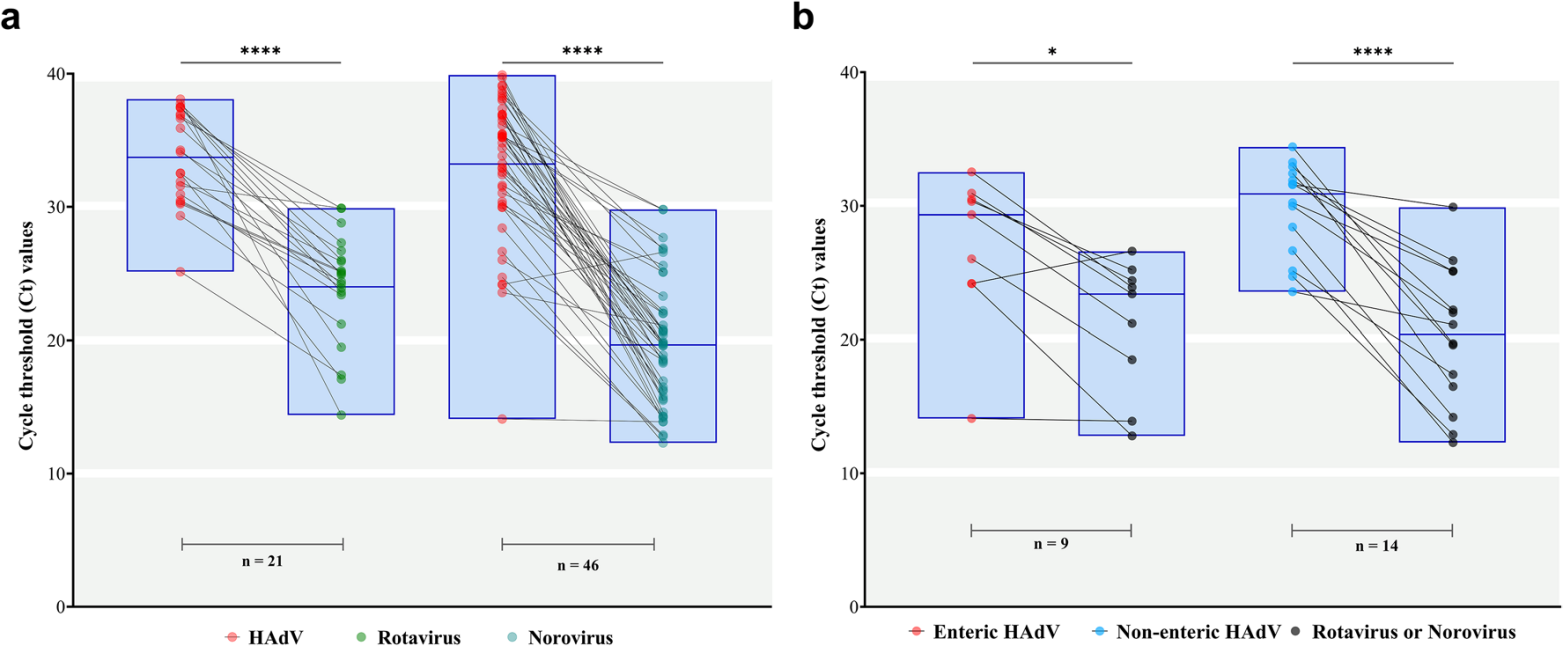
**(a)** Distribution of Cycle Threshold (Ct) values obtained from qPCR for HAdV and by qRT‐PCR for rotavirus and norovirus for each co-detected positive sample. **(b)** Detailed analyses of the Ct values of each characterized enteric and non-enteric HAdV, detected in co-detections with other enteric viruses (rotavirus or norovirus). Box-and-whisker plots show the first and third quartiles (equivalent to the 5th and 95th percentiles), the median (the horizontal line in the box). *p ≤ 0.05; ****p ≤ 0.0001.

Regarding the co-detected samples, we also explored the viral shedding of each sample individually. We identified a general downward trend in the values of Ct comparing HAdV-positive samples co-detected with rotavirus or norovirus. The median Ct values of HAdV- and rotavirus-co-detected samples were 34.1 and 24.9, respectively, and 35 and 19.6 for HAdV- and norovirus, respectively (Fig. 5a). Analysing separately HAdV enteric and non-enteric types co-detected with rotavirus or norovirus yielded similar results.The median Ct values of HAdV enteric types and rotavirus or norovirus were 29.3 and 23.4, respectively, (*p* = 0.0174); while for non-enteric types and rotavirus or norovirus were 30.9 and 20.4, respectively (*p* < 0.0001) (Fig. 5b).

## Discussion

Our 3-year study provides information regarding HAdV incidence, virological and molecular epidemiological features among AGE cases collected from eleven Brazilian states, which account for nearly half of the country’s population (~ 103 million inhabitants. Overall, we detected HAdV in 24.5% of samples from inpatient and outpatient, with a predominance of enteric types, especially type F41 which accounted for a third of total sequenced samples.

In Brazil and elsewhere, there are a limited number of studies in regards to the incidence and molecular epidemiology of HAdV in AGE cases at a national level. In Brazil, some studies performed pre- and post-rotavirus vaccine introduction reported HAdV detection rates ranging from 3.9 to 6.8%, indicating a minor impact of HAdV as an etiologic agent of AGE in Brazil^29–31^. In contrast, but similar to our findings, data from two recent studies conducted in Northern and Southeastern Brazil reported HAdV detection rates of 28.6% and 18.5%, respectively, among children with AGE symptoms^32,33^. Another study in Northern Brazil has detected HAdV in 50% of stool samples from hospitalized children with AGE^34^. Worldwide, HAdV prevalence in AGE stool samples varies greatly, with detection rates ranging from 1.6 to 39.1%, reported from several low- and middle-income countries^22,23,35–39^. Our findings are consistent with reports from China, Iraq, Albania and India that detected HAdV in more than 20% of tested AGE stool samples from children^39–42^. Despite different epidemiological scenarios from different countries, the heterogeneity of data could be attributed to differences in study design, case definition and especially to the diagnostic methods used to detect HAdV. Higher detection rates are often associated with the use of higher-sensitivity molecular assays. As an example, a GEMS study reanalysis with a real-time-based TaqMan Array Card (TAC) revealed a fivefold increase in attributable incidence detection of HAdV types F40/41 when compared to the initially applied enzyme immune assay (EIA)^43^. Another study that reanalysed AGE stool samples using TAC, previously tested with conventional PCR, demonstrated an 18.6-fold increase in the overall detection rate of HAdV and a 4.1-fold increase in the enteric types F40/41^39^.

Regarding age groups, we observed the highest incidence of HAdV among children less than 3 years of age, especially in the group of > 6 to 12 months old, which also showed the highest median viral load across all age groups. Our findings are consistent with large studies performed in several countries (GEMS and MAL-ED reports) that demonstrated the burden of HAdV in young children^44–46^.

The higher detection rate of HAdV in the age group of > 6 to 12 months old could be related to the decline of specific maternal antibodies protection gained from full breastfeeding, which is known to confer some protection against infantile diarrhea^47^. Interestingly, the MAL-ED study demonstrated that full breastfeeding duration was associated with later first acquisition of bacterial and viral pathogens in earlier childhood^48^. Therefore, the decline of maternal antibodies protection and first exposure to viruses may explain the greater HAdV detection and viral load in this age group.

During the first two years of our study, HAdV was detected year-round and no apparent seasonal distribution was observed. These findings are consistent with several long-term studies conducted worldwide, including in Brazil, which did not observe any HAdV seasonal distribution^23,29,30^. From April to September 2020, we observed a dramatic decrease in the number of AGE reported cases, reflecting the impact of COVID-19 pandemic and non-pharmaceutical preventive measures implemented, such as lockdowns, schools and daycares closure, social distancing, heightened hygiene awareness and wearing masks. A multicenter surveillance study conducted with hospitalized pediatric patients in Japan observed a markedly reduction in enteric viruses detection, with a decrease in HAdV detection rate of 13.5% during the COVID-19 pandemic^49^. Other studies have also reported the decline in the incidence of several pediatric infectious diseases of viral etiology during the COVID-19 pandemic, including gastroenteric viruses^50–53^.

A high co-detection rate (30%) of HAdV with rotavirus or norovirus was found during the 3-year period of our study. Two previous studies from our group have demonstrated an overall detection rate of rotavirus and norovirus of 12% and 32%, respectively^27,28^. Given these high detection rates along with the exposure to multiple viruses at similar times increase the likelihood of multiple infections within a short window of time in the same host^54^. Co-detections among enteric viruses are commonly reported, and studies from Brazil, Bangladesh, USA and France have demonstrated the co-detection of HAdV with rotavirus and norovirus, but at lower rates^22,33,55–57^. In addition, Liu et al. (2016)^44^ detected two or more diarrhoea-associated pathogens in almost 40% of 5304 stool samples tested for 32 enteropathogens in the GEMS case–control study with children under the age of five from countries in Africa and Asia. Similarly, during acute respiratory illness, co-viral detection of HAdV with other respiratory viruses is commonly described. A three-year viral surveillance study conducted in Amman, Jordan detected HAdV in 15% of hospitalized children, and over three-quarters had HAdV co-viral detection^58^. Prolonged HAdV shedding following previous infections and the diversity of serotypes and clinical illness might explain the high co-detection rates usually found.

HAdV enteric types F40/41 accounted for nearly half of sequenced samples, with a massive predominance of type F41 compared to type F40. Our data are in line with worldwide findings, which appears to be a global trend in regards to HAdV genotype distribution among AGE cases^22,23,38,57,59^. Previous molecular epidemiological studies conducted in Brazil also demonstrated the predominance of type F41, accounting for more than half of all sequenced samples^29–31,34,60^. A study conducted in Australia, using next generation sequencing technologies, demonstrated a massive predominance of F41 (83.5%) among six major groups of HAdV found in wastewater collected from Sydney and Melbourne treatment plants from 2016 to 2017. In clinical samples, they also found F41 as the most dominant serotype, (52.5% of gastroenteritis cases), followed by C1 and C2^61^. Among the non-enteric HAdV, our study found HAdV species C and B with the second and third highest prevalence, respectively. Previous studies performed in Brazil and other developing countries have reported similar results for non-enteric HAdV, with specie C being the most prevalent and specie A, B and D alternating as the second most prevalent^22,23,29,32,38^. Moreover, despite an increasing number of studies attributing the detection of non-enteric HAdV types in AGE stool samples as a possible gastroenteritis causative agent^13,22,23,40^, the significance of their detection remains unclear. Well-established respiratory-associated-HAdV types are commonly shed for weeks in stool samples^62^. Additionally, some types can establish latency in lymphoid tissues, so a portion of our non-enteric types detection might be linked to the shedding of reactivated latent viruses^63,64^.

Concerning HAdV shedding, few studies have demonstrated HAdV viral load data, particularly comparing with other gastroenteric viruses. In our study, we observed a significantly higher viral load of types 40/41 compared to non-enteric types. As for HAdV co-detections, we found a significantly lower HAdV median Ct value when compared to median Ct values of rotavirus and norovirus. Similarly, a large Canadian case–control study (4,702 samples and 726 positive samples) also reported significantly lower median Ct values (higher viral load) among HAdV-positive cases compared to HAdV-positive controls, as well as for enteric types F40/41 compared to non-enteric^59^. Furthermore, they found significantly lower median Ct values for single detections compared to co-detections. Another case–control multisite study conducted in children under two years old in USA detected a broad range of Ct values for HAdV-positive stool samples and demonstrated a significantly lower median Ct value for AGE cases compared to health controls, especially among samples with HAdV as the only detected pathogen^57^. In contrast, a Chinese case–control study reported no significant difference in viral load shedding between HAdV-positive samples from cases and controls, however, a very small sample size was analyzed, as pointed out as a study limitation by authors^40^. In Brazil, to our knowledge, just one study has analyzed HAdV concentration data and showed significantly lower median viral load among HAdV-positive samples in co-detections compared with rotavirus or norovirus viral loads^31^.

For the enteric HAdV types F40/41, it is well stablished their role as etiological agent of childhood AGE, however, the detection of non-enteric types even in diarrheic samples should be carefully investigated and not routinely be assumed as the causative agent^3^. Thus, the use of sensitive molecular qPCR can improve the HAdV detection in stool samples with the advantage of a reasonable confidence of agent attribution based on low Ct value results. This knowledge, coupled with genotype identification, is important to understand HAdV epidemiology and their burden at a population-level^46,59^.

Our study has some limitations. First, we did not have access to detailed clinical/epidemiological data from patients, which hindered our ability to interpret the detection of HAdV non-enteric types, such as information of respiratory-associated symptoms or recently acute respiratory infections. Second, samples were not screened for additional enteropathogens, such as bacteria, parasites and other gastroenteric viruses that could be involved in AGE clinical cases. Third, we performed PCR typing based on a conserved region of the hexon gene, which could prevent the detection of novel HAdV types and recombinants. For example, we could not distinguish among HAdV species D types based on the amplified region, as the evolution within species D is major related to homologous recombination events rather than nucleotide mutation. Moreover, we were unable to genotype all HAdV-positive samples especially due to higher Ct values, indicating lower viral loads. Finally, we believe that part of HAdV-positive samples may represent prolonged viral shedding following previous infections and might not contribute to AGE in all positive patients, especially in samples with co-viral detections, non-enteric types and low viral loads.

In conclusion, by using a sensitive and quantitative PCR, our three-year surveillance study revealed a high prevalence of HAdV among AGE cases in Brazil, especially the enteric type F41. Molecular epidemiological surveillance of HAdV is essential to monitor the circulation of enteric and non-enteric types and possible emerging strains at a population level, particularly during AGE outbreaks. Moreover, knowledge and continuous monitoring of dominant types is useful for effective vaccine design, as recently proposed by Lee et al. (2020)^3^, in order to reduce the diarrheal disease burden caused by HAdV among young children in low and middle-income countries. Furthermore, besides rotavirus and norovirus that are routinely tested in many countries, the inclusion of qPCR test for enteric types F40/41 is warranted to enhance country-based health epidemiological surveillance programs in order to elucidate the cause of acute diarrheal etiology among infants.

## Materials and methods

### Stool samples

This study included stool samples from children and adults with symptoms of AGE collected between January 2018 and December 2020 from ten states within three Brazilian regions (Southern, Southeastern and Northeastern). AGE was defined as sudden-onset diarrhea (≥ 3 liquid/semi-liquid evacuations in a 24-h period) that may be accompanied by fever, nausea, vomiting, or abdominal pain. All samples were stored at –20 °C until use. HAdV was tested in all samples that tested negative for rotavirus and norovirus received during the period, in addition to ~ 35% of both viruses’ positive samples, to investigate co-detection.

Inpatients and outpatients diarrheic stool samples with epidemiological records were collected by sentinels’ sites at States Central Laboratories and sent to the Laboratory of Comparative and Environmental Virology (LCEV) at Oswaldo Cruz Institute, Fiocruz. The LCEV houses the Rotavirus Regional Reference Laboratory (RRRL) and is part of the ongoing national network for AGE surveillance coordinated by the General Coordination of Public Health Laboratories, Brazilian Ministry of Health. The surveillance is performed through a hierarchical network in which samples are provided by medical request in hospitals and health centers, monitored by the Brazilian Unified Health System (SUS).

### Ethics statements

This study is currently approved by the Ethics Committee of the Oswaldo Cruz Foundation (FIOCRUZ), Brazil (Approval number: CAAE 94144918.3.0000.5248) and was conducted according to the guidelines of the Declaration of Helsinki. Fecal samples were manipulated anonymously and patients’ data were maintained securely. Laboratory activities performed are part of the public health surveillance tasks and Fiocruz Ethics Committee approved the waiver for informed consent.

### Nucleic acid extraction

Viral nucleic acids were purified from 140 μL of clarified stool suspension (10% w/v) prepared with Tris-calcium buffer (pH  7.2). Samples were subjected to an automatic nucleic acid extraction procedure using a QIAamp Viral RNA Mini kit (Qiagen, CA, USA) and a QIAcube automated system (Qiagen), according to the manufacturer’s instructions. The viral nucleic acids extracted were eluted in 60 μL of the elution buffer AVE and immediately stored at –80 °C until the molecular analysis. In each extraction procedure, RNAse/DNAse-free water was used as negative control.

### HAdV detection and quantification

HAdV were detected and quantified by using a TaqMan-based qPCR protocol, with primers and probe targeting the conserved region of the first part of the hexon gene, as previously described^65^. qPCR reactions were performed using 5 µL of the extracted DNA in a final volume of 20 µL, containing 10 µL of 2 × QuantiTect Probe PCR Kit (Qiagen) and primers and probe with final concentrations of 1 µM and 0.25 µM, respectively. Reactions were carried in the Applied Biosystems 7500 Real-Time PCR System (Applied Biosystems, Foster City, CA, USA) with thermal cycling conditions as follows: 2 min at 50 ºC, 15 min at 95 ºC and 40 cycles of 15 s at 95 ºC and 1 min at 60 ºC.

To estimate HAdV viral load, standard curves were prepared using specific viral gBlock Gene Fragments (Integrated DNA Technologies, Coralville, IA, USA) with ten-fold serial dilutions (10^7^–10^0^ genome copies (GC) per reaction) containing the same HAdV amplification region target. Samples were tested for HAdV in duplicate and all samples that crossed the threshold line showing a characteristic sigmoid curve with a cycle threshold (Ct) value < 40 were regarded as positive. All runs included negative and non-template controls (NTC) to ensure the correct interpretation of the results throughout the study. HAdV viral loads were expressed as genome copies per gram (GC/g) of stool specimen.

### Nucleotide sequencing

HAdV-positive samples obtained by qPCR were subjected to conventional PCR targeting a conserved region of the hexon gene using the primer pair hex1deg and hex2deg^66^. The reactions were performed using the Platinum Taq DNA Polymerase enzyme (Invitrogen), following the manufacturer’s recommendations. The expected amplicons of 301 base pairs (bp) were purified using the QIAquick Gel Extraction Kit (Qiagen) following the manufacturer’s recommendation. Sequencing reactions of the purified amplicons were performed using the Big Dye Terminator v. 3.1 Cycle Sequencing Ready Reaction Kit on an ABI Prism 3730 Genetic Analyzer (Applied Biosystems, Foster City, CA, USA) at the Fiocruz Institutional Genomic Platform for DNA sequencing (PDTIS).

### Phylogenetic analysis

Chromatogram analysis and HAdV consensus sequences were obtained using Geneious Prime 2021.1.1 (Biomatters Ltd, Auckland, New Zealand). For HAdV species and type assignment, nucleotide sequences were analyzed in terms of closest homology sequences available in the GenBank database using the Basic Local Alignment Search Tool (BLAST) server (https://blast.ncbi.nlm.nih.gov/Blast.cgi). Phylogenetic trees of the partial hexon gene were constructed using the maximum likelihood method and selected the best-fit evolutionary model for the data set via Kimura two-parameter model (2000 bootstrap replications for branch support) in MEGA X v. 10.2.6^67^, with HAdV reference sequences obtained from the National Center for Biotechnology Information (NCBI) GenBank database. Nucleotide sequences obtained in this study were deposited in the GenBank database (accession numbers: OM470524-OM470634 and OM475617-OM475625).

### Rotavirus and norovirus detection

Rotavirus and norovirus were detected and quantified by using TaqMan-based RT-qPCR protocols. For rotavirus, it was used the primers (NSP3F and R) and probe (NSP3p) targeting the conserved NSP3 gene; and primers pairs (COG1F and R; COG2F and R) and probes (RING1C and RING2), targeting ORF1/2 junction region, were used for norovirus GI and GII detection, respectively. Viral loads were estimated by using standard curves generated from ten-fold serially diluted gBlocks dsDNA fragments containing the target region for each virus. Detailed information on rotavirus and norovirus detection and quantification methods were previously described ^26,27^.

### Statistical analysis

Statistical analyses were performed using GraphPad Prism software v. 9.0.0 (GraphPad Software, San Diego, CA, USA). Mann–Whitney U test was used to assess significant differences between HAdV detection rates, years of collecting samples, viral load values between single and co-detection, age groups and types. Chi-square or Fisher’s exact tests were used for analyzing categorical characteristics in contingency tables. For all analyses, a *p*-value < 0.05 was considered to be statistically significant.

## Data Availability

The datasets generated during the current study are available from the corresponding author on reasonable request. The data are not publicly available due to privacy and ethical restrictions. The datasets generated and/or analyzed during the current study are available in the GenBank repository (accession numbers: OM470524-OM470634 and OM475617-OM475625).
